# Patient and Family Perspective on Transition from Ventricular Access Device to Chest-Sited Port for Intracerebroventricular Infusion in CLN2 Disease

**DOI:** 10.3390/children13030365

**Published:** 2026-03-04

**Authors:** Mahie Gopalka, Jina Patel, Megan Votoupal, Sandi Lam

**Affiliations:** 1 Department of Neurosurgery, Northwestern University Feinberg School of Medicine, Chicago, IL 60611, USA; mgopalka@luriechildrens.org; 2 Division of Pediatric Neurosurgery, Ann & Robert H. Lurie Children’s Hospital, Chicago, IL 60611, USA; jinpatel@luriechildrens.org (J.P.); mvotoupal@luriechildrens.org (M.V.)

**Keywords:** CLN2, cerliponase alfa, intracerebroventricular, ventricular access, caregiver, patient-centered

## Abstract

**Highlights:**

**What are the main findings?**

- Caregiver experience differed meaningfully between scalp-based ventricular access devices and chest-sited ports for intracerebroventricular cerliponase alfa delivery for children with CLN2 disease.
- Chest-sited ports were perceived favorably as access options that supported long-term treatment adherence.

**What are the implications of the main findings?**

- Device and external port selection for intraventricular therapy in CLN2 disease influences caregiver experience, perceived safety, and treatment routines.
- Incorporating caregiver perspectives may improve shared decision-making and patient-centered planning for long-term intracerebroventricular access for patients with CLN2 disease.

**Abstract:**

Background: Cerliponase alfa is currently the only approved disease-modifying therapy for neuronal ceroid lipofuscinosis type 2 (CLN2) disease and requires lifelong intracerebroventricular (ICV) infusion, traditionally via a scalp-sited ventricular access device (VAD). Chest-sited port (chest port) for intracerebroventricular access using a tunneled central venous access device is described as an alternative, though data remain limited. Methods: We present an anonymized caregiver narrative perspective describing two pediatric patients with CLN2 disease receiving cerliponase alfa infusions via different ICV access strategies: one patient who transitioned from a scalp-based ventricular access device to a chest port and one patient who initiated therapy with a chest port. A semi-structured caregiver interview was used to capture experiential insights related to decision-making, procedural burden, safety considerations, and psychosocial adaptation. Results: The caregiver identified key advantages of chest ports for ICV infusion, including durability of the device, enhanced securement, and smooth long-term routine integration. The transition from a scalp VAD to a chest port was described as proactive, well-coordinated, and associated with high caregiver satisfaction. Noted considerations included increased visibility of the access needle to the child, proximity to oral secretions, and potential misidentification of the port by emergency medical services. Families implemented mitigation strategies through labeling, education, and coordination with the care team. Conclusions: This caregiver-centered case report highlights how access device choice meaningfully shapes treatment burden, safety planning, and daily life for families managing CLN2 disease. As chest-port methodologies become adopted, incorporating caregiver and patient perspectives is essential to developing patient-centered treatment options for long-term intracerebroventricular therapy.

## 1. Introduction

Neuronal ceroid lipofuscinosis type 2 (CLN2) disease, a form of Batten disease, is a rare, autosomal recessive pediatric neurodegenerative disease resulting from pathogenic variants in the gene encoding lysosomal enzyme tripeptidyl peptidase 1 (TPP1) [1,2]. Deficiency of TPP1 leads to an accumulation of lysosomal storage material, with subsequent degenerative changes in neurons throughout the central nervous system and retina [3,4]. CLN2 is clinically characterized by seizures, vision loss, speech delay, and psychomotor regression; symptom onset typically occurs between the ages of two and four years [5,6].

Cerliponase alfa, a recombinant form of the human TPP1 enzyme, is the first and only Food and Drug Administration (FDA)-approved treatment for CLN2 disease. This drug is an enzyme replacement therapy; it has shown significant efficacy in slowing disease progression and is considered safe for long-term use [7,8]. Cerliponase alfa is administered into cerebrospinal fluid (CSF) via intracerebroventricular (ICV) infusion every two weeks through an implanted ventricular access device (VAD) placed on the head.

In this approach, a ventricular catheter is inserted into the lateral ventricle, which is connected to a subcutaneous reservoir positioned beneath the scalp that permits repeated percutaneous access for CSF sampling and intraventricular drug delivery [9]. Several reservoir designs exist, most commonly Ommaya and Rickham reservoirs, which function similarly and are collectively referred to here as scalp-based VADs. Although effective, head-sited VAD access requires precise needle placement and may be challenging to maintain during prolonged infusions lasting several hours [10,11]. Patient movement, including seizure activity common in CLN2 disease, can increase the risk of needle dislodgement and compromise sterile technique. In addition, repeated puncture leads to gradual degradation of the reservoir, often necessitating device replacement after several years of use; the integrity of a Rickham reservoir has been estimated to decline after approximately 70–100 punctures.

**Figure 1:**
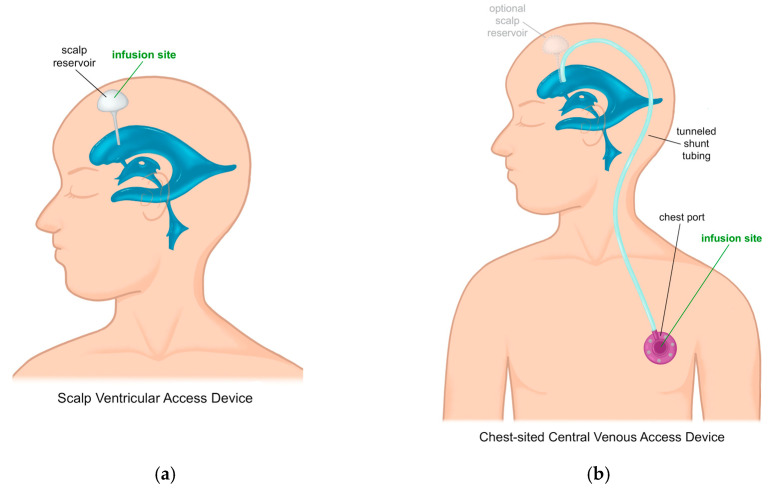
(**a**) Traditional scalp-based ventricular access device used for intraventricular administration of cerliponase alfa, requiring repeated needle access through the scalp. (**b**) Modified access configuration in which a ventricular catheter is connected to a tunneled subcutaneous chest port, allowing intraventricular drug delivery through the chest rather than the scalp. The schematic emphasizes that ventricular access is maintained through the intracranial catheter, while the external access interface differs between scalp-based reservoirs and chest-sited port configurations.

An alternative infusion system has been reported in which a standard ventricular catheter is connected, with or without an intervening ventricular access device, to tunneled shunt tubing to a chest-sited central venous access device port implanted in the anterior chest wall [12,13,14]. This configuration allows intraventricular drug delivery through a chest port rather than repeated scalp puncture (Figure 1). The chest port can be accessed percutaneously; thus, it provides access to the CSF and ICV infusion route for drug infusion.

There is currently limited comparative information between scalp VADs and chest ports for cerliponase alfa infusion. While prior studies have described surgical durability and complication profiles of ventricular access systems used for cerliponase alfa delivery, these reports primarily focus on procedural outcomes rather than caregiver experience. A summary of representative findings from the existing literature is provided in Table 1 to contextualize the present caregiver-centered report.

While these studies primarily evaluate procedural safety and device durability, they provide limited insight into how access configuration shapes caregiver experience and day-to-day treatment adaptation, which is the focus of the present report. Understanding the caregiver perspective can inform ICV access strategies with regard to device placement, treatment options, quality of life, and shared decision-making. This report presents the anonymized caregiver experience of two sibling pediatric patients receiving cerliponase alfa through different access devices: one who transitioned from a scalp VAD to a chest port and one who initiated treatment with a chest port.

## 2. Methods

### Caregiver Interview and Narrative Development

To capture experiential perspectives related to intracerebroventricular access strategies, a semi-structured caregiver interview was conducted with the legal guardian of both patients. The interview was performed by a member of the research team familiar with the clinical care process and focused on decision-making and procedural experience over the course of long-term enzyme replacement therapy.

The interview followed a guided set of prompts designed to elicit narrative descriptions rather than quantitative responses. Interview prompts are available in Supplementary Material S1 to enhance transparency. The interview was recorded and transcribed. The transcript was reviewed by the study authors to identify recurring experiential themes relevant to treatment routines, perceived device advantages and limitations, and caregiver adaptation.

This report is presented as a caregiver-informed clinical case narrative rather than a formal qualitative research study. The intent was to contextualize clinical decision-making and device selection through lived experience within a rare disease setting, complementing existing technical and surgical outcome literature. Observations described in the Results, therefore, reflect caregiver-reported experiences and perceptions rather than objective outcome measurements.

## 3. Results

Results are presented as caregiver-reported experiences derived from the semi-structured interview and are distinguished from objective clinical observations where applicable.

### 3.1. Patient Background

Patient 1 was diagnosed with CLN2 around four years of age after progressive speech delay, seizures, loss of motor skills, and cognitive changes. A VAD was placed, and enzyme replacement therapy began within two weeks of diagnosis.

Patient 2 underwent early diagnostic testing due to family history and was diagnosed during infancy. A chest port connected to a ventricular access device for CSF access and ICV infusion was selected for initial therapy, and enzyme infusions began at nine months of age.

### 3.2. Experience with Scalp Port (Patient 1)

**Figure 2:**
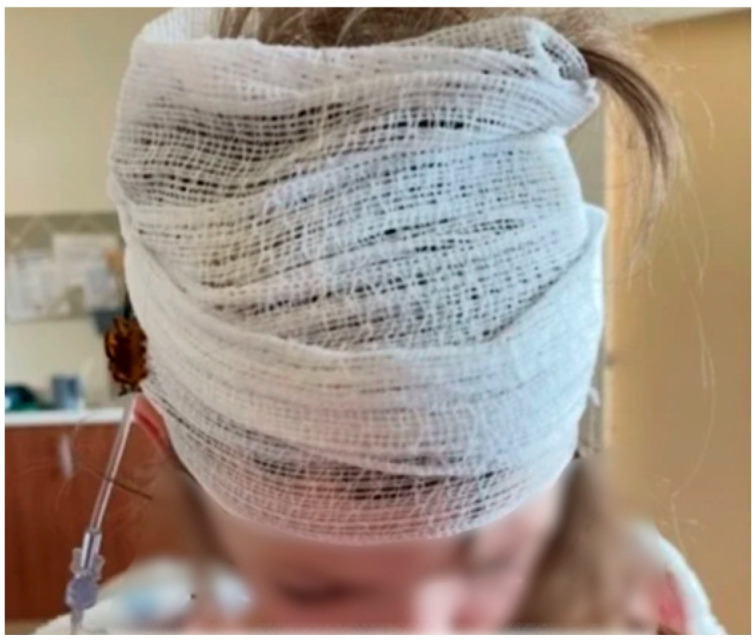
Headwrap (coined “the princess crown” by the caregiver) used to secure the access needle during infusion through a scalp-based ventricular access device. The dressing was used routinely to stabilize the needle during prolonged intraventricular infusions.

Per caregiver interview, Patient 1 began infusions through a scalp reservoir (VAD) placed shortly after diagnosis. The caregiver described an intense adjustment period as the family transitioned from minimal prior medical exposure to highly medicalized, twice-monthly procedures with ICV infusion encounters lasting over 4 h per visit. A headwrap (which the family termed a “princess crown”) was used to secure the infusion needle and became part of a predictable comfort routine (Figure 2).

Accessing the scalp device sometimes required multiple attempts, though most infusions were successful with a single needle access without complications. No topical anesthetic or shaving was used, which the caregiver felt reduced skin irritation and worked well with their care team’s technique.

After 3 years of regular biweekly infusions, the infusion team noted physical signs that the device was aging and had questionable integrity (with a “crunchy” sensation on palpation and access). The caregiver expressed concern about reactive replacement, such as waiting for replacement surgery to be done only if access to an intact VAD was no longer possible. In such a scenario, a VAD with compromised integrity of the access reservoir may have fluid leakage. Such a reactive strategy may risk missed infusions, wasted medication, and potential neurological decline associated with dosing interruptions.

### 3.3. Transition from Scalp to Chest Port (Patient 1)

After a multidisciplinary discussion, the care team and caregiver elected for proactive elective replacement with a chest port. The caregiver described the decision as rooted in: (1) perceived durability of chest ports, which are built for repeated vascular access; (2) desire to minimize brain surgeries over the child’s lifespan; (3) strong trust and shared decision-making with the neurosurgery team; and (4) avoidance of emergency replacement due to device failure.

The transition was described as well-organized and “not panicky,” allowing time for insurance approval and preoperative planning. Postoperative soreness lasted slightly longer than the initial scalp port placement, but the family considered this a minor and acceptable difference. Infusion day logistics changed in subtle ways. The scalp port could be accessed from behind the child. The chest port at the anterior chest wall was accessed by clinicians from the front of the child, making it directly visible. This change took Patient 1 by surprise at first, and it took time to adjust to and become comfortable with the new infusion process. The engaged caregiver contributed to the process by involving more distractions during the access process.

Adhesives such as Tegaderm (3M, Maplewood, MN, USA) and the Stat Lock stabilization device (Becton Dickinson, Franklin Lakes, NJ, USA) were used to attach and secure the needle and tubing to the port during infusions, which caused mild discomfort. Similarly, secretions (saliva or emesis) required increased vigilance due to their proximity to the chest port site.

However, with consistent tape positioning and standardized securement, the care team and family developed a reliable routine that minimized mechanical strain. The ability to see through the secured adhesive tape over the needle access to the chest port and monitor visually for leakage or dislodgement is advantageous. In addition, with the stabilization device securing the connection with the tubing at the anterior chest wall and the ability to place this entire access arrangement under the patient’s clothing, the patients have a range of movement over regular multi-hour infusions. The caregiver expressed “no regrets” regarding the transition, reporting smoother long-term experiences and a strong sense of security with the chest-based device.

### 3.4. Initiating Therapy with a Chest Port (Patient 2)

Patient 2 received a chest port initially due to perceived securement advantages given the patient’s relatively young age (under 1 year old) at the time of treatment initiation. The caregiver reported their partner being uncertain about the chest port, given that the older sibling had a scalp VAD access. However, the family felt that their trust in the care team and the open discussion with the neurosurgeon about the mechanics of the chest port allowed them to feel comfortable choosing a chest port for Patient 2.

The caregiver described being able to safely swaddle, hold, and soothe Patient 2 while maintaining secure tubing during infusions, which would not have been possible with a scalp access site. One early episode of accidental tugging led to a minor leak, after which the care team consistently used Stat Lock securement devices for added stability. As Patient 2 grew to be verbal and interactive, coping strategies and routines required periodic modification. During certain developmental phases, brief use of intranasal anxiolytics helped facilitate access. Over time, infusions became part of Patient 2’s identity and routine. The caregiver reported high satisfaction with the chest-port access and expressed a desire to highlight positive aspects of this approach with the broader CLN2 community.

### 3.5. Safety and Practical Considerations

The caregiver identified a safety concern that her family and others in the CLN2 community have discussed regarding chest ports. There is a possible risk that emergency medical services (EMS) providers may mistakenly assume a chest port is a vascular access device to deliver drugs to the bloodstream rather than directly into CSF by ICV access. As a result, this family implemented clear labeling on mobility equipment (car seats, car doors) and explicit instructions in school and care plans. The caregiver also lives in a smaller community and reached out to local EMS providers for direct communication with them to mitigate risks for the children.

This caregiver shared advice from the broader CLN2 community. Examples such as labeled bracelets or adhesive temporary tattoos near the chest port illustrate a diversity of approaches. Additional community concerns included chest port placement relative to breast tissue in older children and variable port positioning across centers, which may limit generalizability for comfort and taping strategies. However, the caregiver shared that these concerns were quickly and systematically eased by her care team for her children.

### 3.6. Comparative Treatment Timeline

To contextualize the caregiver experience across both access strategies, Table 2 presents a simplified timeline of major treatment milestones for each patient. This timeline reflects how device decisions and treatment adaptation were experienced longitudinally by the family.

## 4. Discussion

### 4.1. Decision-Making Drivers

Across both patients, caregiver perspectives highlight that durability and proactive replacement timing are central to device-choice decisions if given the choice. A strong partnership with the neurosurgical team played a critical role in minimizing anxiety and supporting informed decision-making. Community narratives influenced expectations, particularly stories about device failures with the scalp port, leading to enzyme loss during infusions and urgent device replacements. Proactive replacement and switching to the chest port mitigated many of these concerns for this family.

The timeline presented in Table 2 above illustrates how the access strategy influenced lived treatment experience across developmental stages within a single-family context.

### 4.2. Clinical and Procedural Differences

From the caregiver’s perspective, reported advantages of chest ports included (1) stable securement strategies, especially for younger children; (2) expected durability of chest ports over ventricular access devices; (3) relative ease of maintaining sterility and reduced interaction with hair; (4) avoidance of hair-bearing areas, eliminating the question of shaving or negotiating hair parting; and (5) perception of lower risk of unexpected device failure.

Reported considerations included (1) greater visibility of the needle to the child during access; (2) proximity to oral secretions or reflux; (3) potential confusion by EMS or unfamiliar clinicians; and (4) unfamiliarity and lack of available information regarding chest ports.

### 4.3. Psychosocial Adaptation

The caregiver described a deliberate reframing of biweekly multi-hour infusions as routine “self-care,” using consistent sensory cues (music and scent) and stable routines to normalize twice-monthly infusion procedures. Each patient adapted differently based on personal temperament and developmental stage. The family’s experience underscores the importance of integrating child life specialists, maintaining predictable rituals, and preparing families for individualized adjustment processes.

### 4.4. Importance of Parents’ Voices

While surgical feasibility, complication rates, and device longevity are essential considerations in selecting an intraventricular access strategy, these metrics alone do not capture the burden of treatment for families living with CLN2 disease [13,14]. Caregivers shoulder the responsibility of navigating repeated infusions, coordinating emergency preparedness, managing psychosocial stress, and sustaining long-term adherence in the context of a progressive neurodegenerative illness. This report elevates caregiver experience as a form of experiential expertise, offering insights that are not readily captured in retrospective or prospective surgical outcomes, device survival analyses, or pharmaceutical reports. By documenting lived experiences across two different access strategies within the same family, this case report provides nuanced, comparative insights into routine formation, safety concerns, emotional adaptation, and trust-building with care teams. Incorporating caregiver voices alongside clinical outcomes is critical to developing truly patient-centered strategies and shared decision-making for intracerebroventricular access in CLN2 disease. These observations represent experiential insights rather than measured clinical outcomes and are intended to complement, rather than replace, existing surgical outcome literature.

### 4.5. Community-Level Implications

The lack of published patient-facing data on chest ports leads to heavy reliance on anecdotal community stories, which can generate fear-based narratives. From a caregiver perspective, she emphasized the need for (1) standardized educational materials for families; (2) guidance for EMS and school systems; (3) clearer clinical decision support for proactive replacement; (4) broader dissemination of real-world experiences; and (5) quantification of potential quality, safety, and value benefits if fewer replacements reduce cost and surgical burden.

## 5. Conclusions

This anonymized case report highlights the lived experiences of a caregiver managing two different ICV access strategies in siblings for cerliponase alfa infusion in CLN2 disease. The transition from a scalp VAD to a chest port for Patient 1 and the initial use of a chest port for Patient 2 collectively illustrate advantages related to durability, securement, and long-term routine integration. Chest ports also carry unique considerations, including oral secretion management and the risk of misidentification by emergency responders. These perspectives provide valuable insight into decision-making, treatment burden, and psychosocial adaptation in CLN2 care from a caregiver and family perspective. They underscore the importance of proactive planning, multidisciplinary communication, and the incorporation of patient and caregiver voices into patient-centered shared decision-making for intracerebroventricular access device selection.

## Figures and Tables

**Table 1:** Selected published reports on intracerebroventricular access strategies for cerliponase alfa delivery in CLN2 disease.

Study	Access Strategy Evaluated	Design/Cohort	Key Reported Device-Related Findings
Craven et al. (2022) [10]	Head-sited ventricular access devices (VADs)	Single-center survival analysis of VAD insertions/revisions	Reported median survival of newly inserted VADs of 2317 days was limited by repeated puncture; the main reason for revision was suspected infection.
Read et al. (2025) [14]	Chest-sited vs. head-sited ICV access devices	Single-center comparative experience evaluating safety/efficacy/user experience	Evaluated safety, efficacy, and user experience comparing chest-sited versus head-sited devices (details summarized in source article).
Boop et al. (2025) [13]	Chest-port system connected to intraventricular access	Case series	Reported 7 patients undergoing placement or conversion to a CVAD chest-port system; authors conclude the system supports long-term infusion and may be better tolerated than scalp-based infusions.

**Table 2:** Longitudinal treatment timeline illustrating major clinical milestones and access device transitions for both patients.

Treatment Milestone	Patient 1	Patient 2
Diagnosis (in the same month)	Genetic diagnosis at age 4 years guided by clinical condition	Presymptomatic genetic diagnosis at age 9 months following a sibling’s diagnosis
Initial access surgery	Scalp VAD placement	Chest port placement
Start of cerliponase alfa	Initiated within 3 weeks of diagnosis	Initiated within 1 month of diagnosis
Early infusion phase	Scalp access with a successful securement routine for 3 years	Chest port access integrated into infant care
Revision surgery	Elective transition to chest port toward end-of-service integrity of VAD	None
Post-transition course	Stable chest-port infusions for the subsequent 2 years	Stable chest-port infusions throughout 5 years
Status at interview (September 2025)	Caregiver reports satisfaction with established routine	Caregiver reports satisfaction with established routine

## Data Availability

The anonymized data presented in this study are available on request from the corresponding author due to the privacy of the patients, caregivers, and family.

## Supplementary Materials

The following supporting information can be downloaded at https://www.mdpi.com/article/10.3390/children13030365/s1. File S1: Structured Interview Guiding Questions

## Abbreviations

The following abbreviations are used in this manuscript:

CLN2	Neuronal ceroid lipofuscinosis type 2
ICV	Intracerebroventricular
VAD	Ventricular access device
TPP1	Tripeptidyl peptidase 1
FDA	Food and Drug Administration
CSF	Cerebrospinal fluid
