# Supplementary material for: Patient and Family Perspective on Transition from Ventricular Access Device to Chest-Sited Port for Intracerebroventricular Infusion in CLN2 Disease
Source: Children (Basel). 2026 Mar 4;13(3):365. doi: 10.3390/children13030365 (PMC13025636; doi:10.3390/children13030365)
Supplement: Supplementary file 1 [file children-13-00365-s001.zip › children-4161296-supplementary.pdf]

1. Diagnostic and Early Treatment Experience

- Can you walk me through the diagnostic journey for your daughters?
- How did you first learn about Brineura, and what were your initial thoughts or feelings about the treatment?
- What were your concerns (if any) when starting the scalp infusions?

2. Experience with Scalp Infusions

- What aspects of the scalp infusions were challenging—emotionally, logistically, or medically?
- Did your daughters experience anxiety or discomfort during or around infusion days?
- How did you manage the routine of scalp infusions at home and at the hospital?

3. Infusion Logistics & Integration into Family Life

- How do you fit Brineura infusions into your family's life and routines?
- What are infusion days like for your family from start to finish? (e.g., preparing, travel, time in the hospital, returning home)
- How do you manage care for both children on the same day?
- How has this biweekly schedule impacted your work, childcare, or other responsibilities?
- Have you developed any strategies or routines that make infusion days more manageable?

4. Infusion Day Experience

- Can you describe what a typical infusion day looks like at the hospital?
- How do you and the healthcare team work together to ensure things go smoothly?
- Are there particular steps during the infusion process that you find especially stressful (e.g., placement of the needle, length of infusion, verifying flow into the brain)?
- Do you ever worry about the needle or tubing being dislodged or malfunctioning during the infusion?
- How do your daughters typically respond emotionally or physically during the infusion process?

5. Decision-Making Process Around Switching to CVAD

- Who first introduced the idea of switching from scalp infusions to a central venous access device (CVAD)?
- What factors played the biggest role in your decision (e.g., infection risk, comfort, pain, time in hospital)?
- Did you have any hesitations about moving forward with the CVAD placement?

6. Experience After Switching to CVAD

- How has the infusion process changed since transitioning to the CVAD?

- Have you noticed any differences in your daughters' comfort, behavior, or emotional responses?
- How has the experience been in terms of port maintenance, home care, or complications?
- Has the switch affected your hospital visits or your workflow on infusion days?

7. Broader Impacts

- How has this change impacted your family's day-to-day life or care coordination?
- Do you feel the switch has influenced your daughters' overall quality of life?
- Has it changed your relationship or communication with your healthcare team?

8. Retrospective Evaluation

- Looking back, do you feel the switch to a CVAD was the right decision for your family?
- Would you recommend this option to other families considering the change?
- Is there anything you wish you had known earlier, or anything you would have done differently?
